# Aggregate index of systemic inflammation as a novel prognostic biomarker in Chinese patients with acute decompensated heart failure: a population-based real-world study

**DOI:** 10.3389/fendo.2025.1627821

**Published:** 2025-08-13

**Authors:** Lin Hu, Yangjie Deng, Chuanjin Liu, Yinghao Kuang, Xinfang Huang, Jinyan Zhang, Wanfen Huang, Yafei Jian, Guobo Xie, Yang Zou, Shuhua Zhang

**Affiliations:** ^1^ Department of Cardiology, Jiangxi Provincial People’s Hospital, The First Affiliated Hospital of Nanchang Medical College, Nanchang, Jiangxi, China; ^2^ Department of Cardiology, Yongfeng County Hospital of Traditional Chinese Medicine, Jian, Jiangxi, China; ^3^ Jiangxi Cardiovascular Research Institute, Jiangxi Provincial People’s Hospital, The First Affiliated Hospital of Nanchang Medical College, Nanchang, Jiangxi, China

**Keywords:** inflammation, aggregate index of systemic inflammation, acute decompensated heart failure, Chinese, risk stratification inflammation, risk stratification

## Abstract

**Introduction:**

Inflammation is hypothesized as an early trigger for decompensation in heart failure patients. This study aims to evaluate the prognostic value of a novel inflammatory biomarker, the Aggregate Index of Systemic Inflammation (AISI), for predicting 30-day mortality in patients with acute decompensated heart failure (ADHF).

**Methods:**

This analysis included 2,765 patients from the Jiangxi-ADHF II registry (2018-2024). Complete blood counts were measured at hospital admission, with 30-day mortality outcomes followed. Multivariable Cox proportional hazards model was employed to analyze the association between AISI and all-cause mortality.

**Results:**

During 30-day follow-up, the overall mortality rate was 7.34% (203 deaths), with rates progressively increasing across AISI quartiles (Q1-Q4: 2.32%, 3.33%, 5.21%, 18.50%). Compared with the lowest AISI quartile, the highest quartile was associated with a 210% higher risk of 30-day mortality (Hazard Ratio: 3.10, 1.62-5.94). This association remained robust across multiple sensitivity analyses, including subgroup analysis, temporal sensitivity assessments, and data integrity verification. Further spline regression analysis revealed a U-shaped curve association between AISI (and LnAISI) and 30-day mortality in ADHF patients (P for non-linearity < 0.05). In general, both extremely low and high levels of AISI and its natural logarithm (LnAISI) were associated with an increased risk of 30-day mortality in ADHF patients. Moreover, in predicting 30-day mortality among ADHF patients, the AISI demonstrated significantly superior predictive value compared to white blood cell count, neutrophil count, monocyte count, and lymphocyte count (Area under the curve=0.77; all DeLong tests P <0.05), with an optimal threshold of 925.44.

**Discussion:**

This population-based retrospective cohort study demonstrated the predictive value of AISI for short-term outcomes in Chinese ADHF patients. Compared to conventional inflammatory biomarkers, AISI significantly improved the predictive performance for 30-day mortality in ADHF patients. These findings may facilitate optimized prevention of adverse outcomes in ADHF and enable early risk stratification through targeted assessment of individual ADHF patients.

## Graphical Abstract

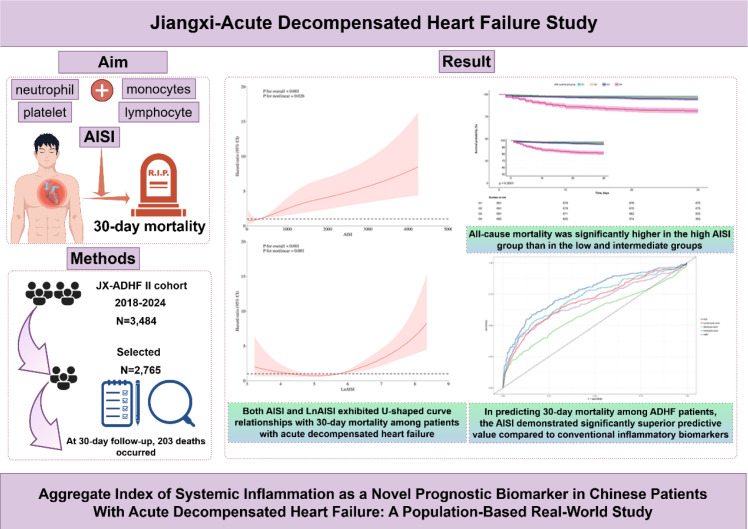

## Background

Acute decompensated heart failure (ADHF) refers to the abrupt deterioration of cardiac function, which may occur *de novo* or as a worsening of pre-existing chronic heart failure due to various underlying etiologies. Pathologically, it is characterized by volume overload and hemodynamic derangement, while clinically, it manifests with symptoms such as acute dyspnea, peripheral edema, and fatigue (1–4). Despite recent advancements in ADHF management, such as the utilization of mechanical circulatory support devices, heart transplantation, and the development of novel pharmacological agents, overall improvements in clinical outcomes remain modest (5, 6). Studies have shown that approximately 25% of ADHF patients are readmitted within 30 days after discharge, and the 30-day mortality rate approaches 10% (1, 7, 8). This imposes a significant economic and healthcare burden on patients, their families, and society. More critically, low- and middle-income countries face an even greater disease burden due to uneven regional distribution of healthcare resources (9). Therefore, early risk stratification using accessible and effective biomarkers may help improve short-term clinical outcomes in hospitalized patients with ADHF.

The pathophysiological manifestations of ADHF are pleiotropic, primarily influenced by multiple factors such as the type of cardiac dysfunction, extent of ventricular involvement, vascular tone regulation, neurohormonal/inflammatory activation status, and comorbid conditions (10). These factors also constitute key targets for assessment and monitoring in the clinical management of ADHF (6, 10). Investigations into ADHF pathophysiological progression reveal intimate linkages with dysregulated activation of the neurohormonal system and inflammatory pathways (11–13). Notably, elevated inflammatory marker levels in ADHF patients often precede elevations in neurohormonal biomarkers like N-Terminal Pro-Brain Natriuretic Peptide (NT-proBNP) (14). This temporal pattern implies inflammation may act as an initial trigger for decompensation. To date, substantial clinical evidence has been collected confirming the involvement of inflammation in the pathogenesis and progression of ADHF (12, 13, 15). Additionally, various inflammatory biomarkers (including neutrophil, monocyte, and lymphocyte counts) have been investigated for their potential utility as diagnostic and prognostic indicators in ADHF (13, 15, 16). However, predictive models relying solely on blood cell counts have consistently shown limited prognostic value (13, 15–17). The Aggregate Index of Systemic Inflammation (AISI), a novel inflammatory marker developed recently using hematological parameters, provides a composite assessment of systemic inflammatory status through integration of neutrophil, monocyte, platelet (PLT), and lymphocyte counts (18). Accumulating evidence supports the clinical utility of AISI for risk stratification and prognosis prediction across multiple disease categories, including metabolic disorders (19–22), immune-mediated diseases (23–25), critical care scenarios (17, 26, 27), and psychiatric illnesses (28, 29), and its clinical utility generally surpasses that of traditional inflammatory markers. Additionally, the application of the AISI in cardiovascular diseases (CVD) has attracted growing attention. Emerging evidence indicates that elevated AISI levels are associated with an increased risk of various CVDs, including HF (30–34), and may serve as a predictor of poor prognosis in patients with hypertension and acute myocardial infarction (35, 36). However, the prognostic significance of the AISI in HF patients remains unclear. To address this gap, the present study aimed to investigate the association between AISI and 30-day mortality in ADHF patients, using data from the Jiangxi-ADHF II (JX-ADHF II) cohort.

## Methods

### Study data

The JX-ADHF study is a population-based longitudinal cohort study (37) conducted in Jiangxi Province from 2018 to the present. In the current analysis, we utilized data from the JX-ADHF II cohort (2018-2024). This cohort systematically collected comprehensive baseline clinical characteristics [gender, age, New York Heart Association (NYHA) functional class at admission], lifestyle habits (smoking and drinking status), comorbidities [hypertension, diabetes, stroke, coronary heart disease (CHD)], echocardiographic data (left ventricular ejection fraction, LVEF), blood test data, and 30-day follow-up outcomes for ADHF patients admitted to Jiangxi Provincial People’s Hospital between January 2018 and January 2024. It should be noted that blood samples were collected within the first 24 hours of hospital admission for ADHF patients, including biochemical parameters [triglyceride (TG), total cholesterol, high-density lipoprotein cholesterol (HDL-C), low-density lipid cholesterol (LDL-C), creatinine (Cr), alanine aminotransferase (ALT), aspartate aminotransferase (AST), uric acid (UA), fasting plasma glucose (FPG), analyzed by HITACHI LAbOSPECT 008 automated biochemical analyzer], Complete blood count parameters [white blood cell count (WBC), red blood cell count, PLT, neutrophil count, lymphocyte count, and monocyte count, analyzed by Sysmex XN-3000 hematology analyzer], and the cardiac biomarker NT-proBNP.

The JX-ADHF II cohort underwent rigorous data integrity and quality checks at the Science Education Department of Jiangxi Provincial People’s Hospital and the Jiangxi Cardiovascular Institute. Regarding the use of research data, the JX-ADHF project team strictly adhered to ethical review requirements. The study analysis was approved by the Ethics Committee of Jiangxi Provincial People’s Hospital (approval no: 2024-01) and obtained authorization from patients and their families.

### Study population

**Figure 1:**
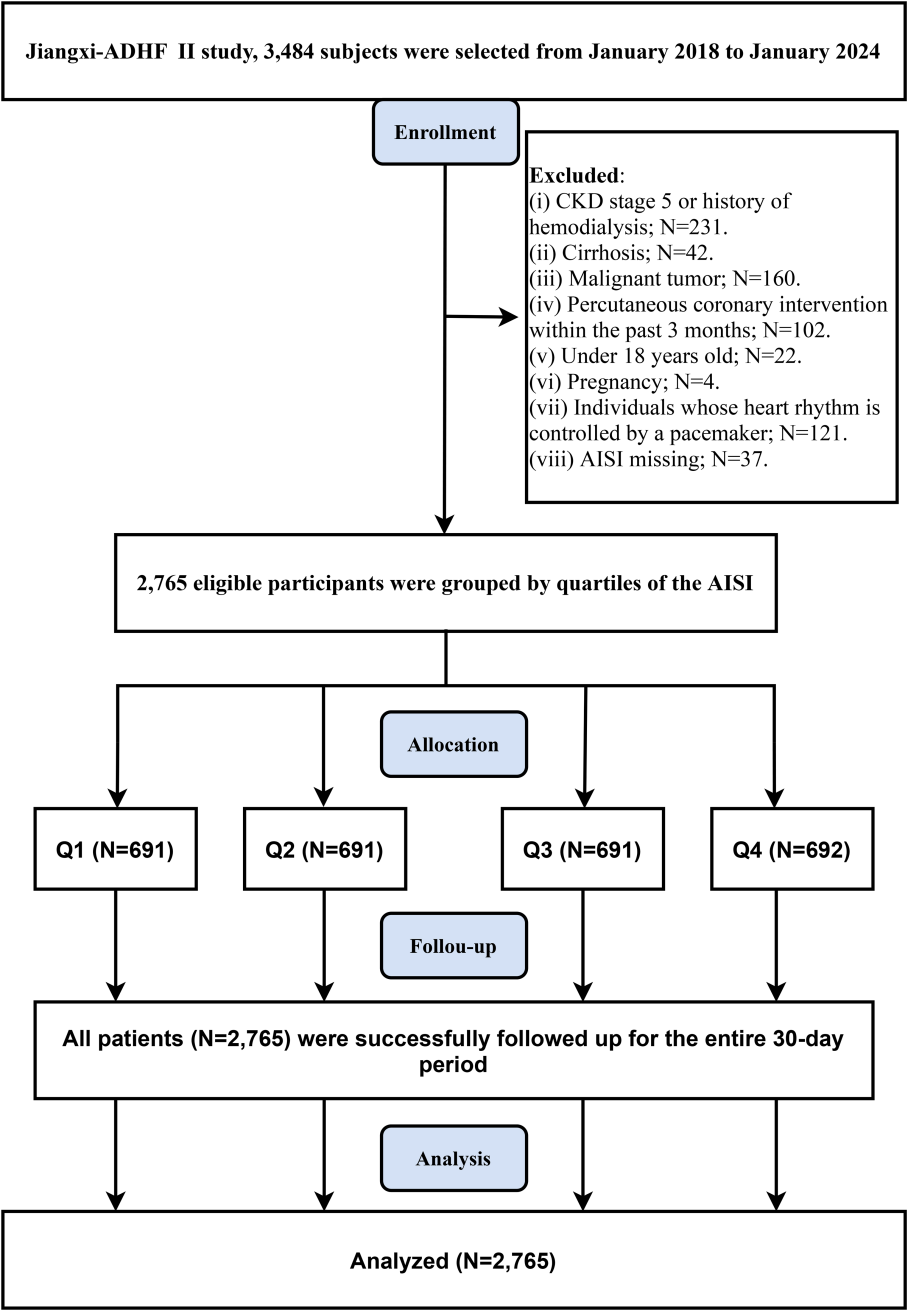
Flow chart of participant inclusion/exclusion in this retrospective cohort study. AISI, Aggregate Index of Systemic Inflammation; ADHF, acute decompensated heart failure; CKD, Chronic kidney disease.

The JX-ADHF II cohort included 3,484 patients with ADHF. Based on the study design, the research population was screened using the following criteria (**Figure 1**): (1) Patients with liver cirrhosis, uremia, or chronic kidney disease undergoing hemodialysis were excluded (n=42 + 231), as non-cardiac water-sodium retention could confound cardiac function and short-term outcomes. (2) HF patients who underwent percutaneous coronary intervention recently were excluded (n=102), as reperfusion therapy may influence short-term prognosis. (3) Patients with pacemaker implantation were excluded (n=121), as their heart rhythms were considered independent of autonomic nervous control. (4) Patients with malignant tumors were excluded (n=160), given the potential strong impact of malignancies on short-term survival. (5) Pregnant patients (n=4) or minor patients (n=22) were excluded. (6) Individuals with missing AISI data were excluded (n=37). Finally, 2,765 ADHF patients were included in the final study analysis.

### Calculation formula


$$\begin{array}{c} \;\text{AISI }=\;(\text{neutrophil count }\times \text{ platelet count}\;\;\\ \times \text{ monocyte count})\;/\text{ lymphocyte count} \end{array}$$

Units: The values are calculated using absolute counts (×10^9^/L).

Note: Due to the non-normal distribution of AISI (**Supplementary Figure 1**) and its wide numerical range (5th-95th percentile: 67.22-2708.04), the effect of a per-unit increase on the dependent variable might be relatively small, potentially leading to lower absolute values of regression coefficients. To address this limitation and enhance the interpretability of clinical outcomes, we performed a natural logarithm (ln) transformation on AISI prior to regression analysis (16, 18).

### Study outcome

The primary outcome in this study was all-cause mortality within 30 days post-admission for ADHF. Day 0 was defined as the hospitalization date for survival duration calculations. Patients were followed daily for 30 days post-admission to monitor survival status, with outcome dates recorded accordingly.

### Missing data handling

The missingness patterns for covariates in this study are summarized in **Supplementary Table 1**, which revealed partial missing data for LVEF, ALT, AST, Cr, UA, and blood lipid/glucose parameters. We further compared baseline characteristics between individuals with missing and non-missing data, demonstrating that the missingness in this study conformed to the missing completely at random assumption (**Supplementary Table 2**). Given the relatively small overall missing proportion and the missing completely at random pattern, we conducted primary analyses using complete-case data to preserve the original data structure, while employing multiple imputation in sensitivity analyses to verify result robustness.

### Statistical analysis

A two-tailed significance level of 5% was adopted. All analyses were performed using Empower (R) (version 2.0) and R language (version 3.4.1). Demographic and clinical features were summarized using appropriate descriptive statistics [categorical: counts (%); normally distributed continuous: mean ± standard deviation; skewed distributed continuous: median (interquartile ranges)]. Analytical approaches were tailored to variable types and distributions for comparative analyses.

**Figure 2:**
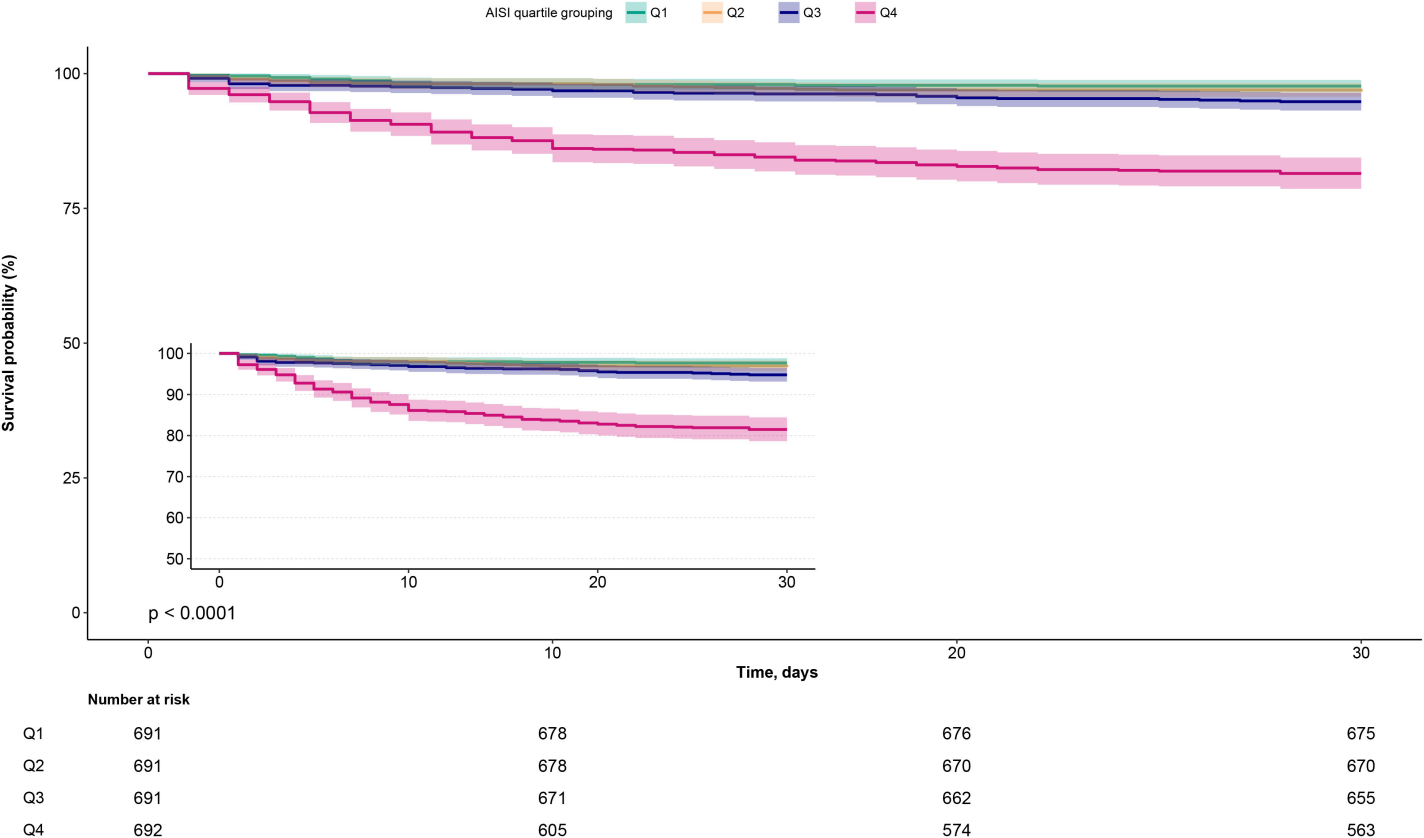
Kaplan-Meier survival curves stratified by AISI quartiles in ADHF patients. ADHF, acute decompensated heart failure; AISI, Aggregate Index of Systemic Inflammation.

The temporal analysis of 30-day mortality included the following steps: Firstly, cumulative survival rates were analyzed across AISI quartile groups using Kaplan-Meier estimation and log-rank testing. Subsequently, a multivariable Cox proportional hazards model was employed to evaluate the association between AISI and 30-day mortality, adjusting for potential confounding factors affecting dependent and independent variables. These confounders encompassed baseline clinical characteristics (gender, age, NYHA classification), lifestyle habits (smoking and drinking status), comorbidities (hypertension, diabetes, stroke, CHD), cardiac function assessment indicators (LVEF and NT-proBNP), red blood cell count, hepatorenal function markers (AST, Cr), UA, and lipid/glucose metabolism parameters (total cholesterol, TG, HDL-C, LDL-C, FPG). Notably, all included confounders passed collinearity screening among covariates (**Supplementary Table 3**). Additionally, the proportional hazards assumption was validated through visual inspection of survival curves across AISI groups (**Figure 2**).

The restricted cubic spline (RCS) analysis was conducted after establishing the associations of AISI and LnAISI with 30-day mortality, aiming to visualize their dose-response relationship. When significant non-linear associations were detected (38), a recursive algorithm was applied to identify inflection points where the relationship changed, followed by piecewise Cox regression to quantify hazard ratios (HRs) and 95% confidence intervals (CIs) before and after these inflection points.

Several subgroups were constructed to examine whether the association between LnAISI and 30-day mortality exhibited population-specific heterogeneity. Subgroup factors included age (stratified by median), gender, LVEF, NYHA classification, and comorbidity status. Heterogeneity within subgroups was assessed using the likelihood ratio test.

Our investigation utilized receiver operating characteristic curve analysis to assess the mortality prediction performance of both AISI and conventional inflammatory indices (neutrophil, lymphocyte, and monocyte counts, along with WBC). Predictive accuracy was quantified through the area under the curve (AUC) determination, with complementary measures including sensitivity, specificity, and optimal cutoff values. Furthermore, we investigated the predictive performance of adding AISI to classic HF indicators (NT-proBNP and LVEF) for 30-day mortality, and compared the AUC values using the DeLong test (39).

### Sensitivity analysis

1. To minimize the influence of frailty on study outcomes, we defined a frail subgroup as patients with ≥3 comorbidities. The association analyses were repeated after excluding this potentially frail population.
2. Addressing potential reverse causality, we implemented a 3-day survival threshold for study inclusion, followed by validation through repeated association testing in this refined cohort.
3. Given that acute inflammation might influence AISI and study outcomes, we conducted a sensitivity analysis by excluding patients with baseline pulmonary infection and replicated the primary analysis procedures.
4. To address potential bias introduced by missing data, we employed the multiple imputation by chained equations method under the fully conditional specification framework to impute missing values. Specifically, for each variable with missing data, an individual imputation model was fitted. Furthermore, we examined the distributions of missing variables before and after imputation using density plots (**Supplementary Figure 2**), which demonstrated that the imputed data closely resembled the original distributions. Based on these results, we repeated the primary analysis workflow using the imputed dataset.

## Results

### Baseline characteristics of the study population

The current analysis included 2,765 ADHF patients, with a mean age of 69 years and a male-to-female ratio of 1.38:1. We summarized the baseline characteristics of the study population according to AISI quartiles (**Table 1**). Baseline data revealed that higher AISI groups had significantly greater proportions of males and patients with comorbid hypertension, diabetes, stroke, CHD, as well as those with smoking status and NYHA class IV. Additionally, patients with elevated AISI levels were generally older and exhibited higher levels of WBC count, neutrophil count, monocyte count, PLT, ALT, AST, Cr, UA, TG, LDL-C, FPG, and NT-proBNP, alongside lower levels of HDL-C and lymphocyte count.

**Table 1:** Summary of baseline characteristics of the study population according to AISI quartiles group.

Variable	AISI quartiles				*P*-value
	Q1	Q2	Q3	Q4	
No. of subjects	691	691	691	692	
Age (years)	69.00 (59.00-77.00)	70.00 (60.00-78.00)	70.00 (60.00-79.00)	74.00 (64.00-81.00)	<0.01
Gender (n,%)					<0.01
Male	354 (51.23%)	390 (56.44%)	426 (61.65%)	433 (62.57%)	
Female	337 (48.77%)	301 (43.56%)	265 (38.35%)	259 (37.43%)	
Hypertension (n,%)					<0.01
No	439 (63.53%)	372 (53.84%)	375 (54.27%)	357 (51.59%)	
Yes	252 (36.47%)	319 (46.16%)	316 (45.73%)	335 (48.41%)	
Diabetes (n,%)					<0.01
No	562 (81.33%)	530 (76.70%)	488 (70.62%)	475 (68.64%)	
Yes	129 (18.67%)	161 (23.30%)	203 (29.38%)	217 (31.36%)	
Stroke (n,%)					0.02
No	595 (86.11%)	582 (84.23%)	577 (83.50%)	554 (80.06%)	
Yes	96 (13.89%)	109 (15.77%)	114 (16.50%)	138 (19.94%)	
CHD (n,%)					<0.01
No	535 (77.42%)	445 (64.40%)	454 (65.70%)	445 (64.31%)	
Yes	156 (22.58%)	246 (35.60%)	237 (34.30%)	247 (35.69%)	
NYHA classification (n,%)					<0.01
III	520 (75.25%)	495 (71.64%)	465 (67.29%)	365 (52.75%)	
IV	171 (24.75%)	196 (28.36%)	226 (32.71%)	327 (47.25%)	
Drinking status (n,%)					0.77
No	628 (90.88%)	628 (90.88%)	623 (90.16%)	619 (89.45%)	
Yes	63 (9.12%)	63 (9.12%)	68 (9.84%)	73 (10.55%)	
Smoking status (n,%)					0.01
No	599 (86.69%)	573 (82.92%)	579 (83.79%)	555 (80.20%)	
Yes	92 (13.31%)	118 (17.08%)	112 (16.21%)	137 (19.80%)	
LVEF (%)	47.22 (12.49)	46.06 (11.78)	45.28 (12.21)	46.95 (11.62)	0.01
WBC (×10^9^/L)	4.72 (1.36)	5.84 (1.50)	7.03 (1.96)	10.52 (4.30)	<0.01
Neutrophil count (×10^9^/L)	2.80 (2.30-3.40)	3.80 (3.20-4.50)	4.90 (4.15-5.80)	7.79 (6.00-10.40)	<0.01
Lymphocyte count(×10^9^/L)	1.29 (0.90-1.62)	1.16 (0.81-1.60)	1.06 (0.80-1.48)	0.78 (0.50-1.13)	<0.01
Monocyte count (×10^9^/L)	0.36 (0.30-0.42)	0.46 (0.38-0.57)	0.55 (0.46-0.70)	0.70 (0.52-0.94)	<0.01
RBC (×10^12^/L)	4.01 (0.78)	4.07 (0.73)	4.12 (0.80)	4.01 (0.81)	0.02
PLT (×10^9^/L)	128.00 (96.50-160.00)	161.00 (133.00-198.00)	184.00 (146.00-227.00)	208.00 (164.00-266.00)	<0.01
ALT (U/L)	20.00 (13.00-31.00)	21.00 (14.00-34.00)	23.00 (14.00-43.00)	23.00 (14.00-47.00)	<0.01
AST (U/L)	26.00 (19.00-35.00)	25.00 (19.00-36.00)	26.00 (19.00-41.00)	29.50 (20.00-51.00)	<0.01
Cr (umol/L)	81.00 (66.00-104.00)	87.00 (68.00-119.00)	96.00 (71.75-129.00)	104.00 (79.00-169.00)	<0.01
UA (umol/L)	402.00 (329.00-499.00)	418.00 (336.00-528.50)	441.50 (349.00-554.25)	456.00 (348.00-589.75)	<0.01
TG (mmol/L)	1.05 (0.81-1.42)	1.16 (0.86-1.52)	1.14 (0.90-1.60)	1.22 (0.92-1.62)	<0.01
TC (mmol/L)	3.64 (3.05-4.36)	3.80 (3.16-4.45)	3.76 (3.10-4.42)	3.78 (3.18-4.51)	0.06
HDL-C (mmol/L)	1.00 (0.81-1.19)	0.99 (0.80-1.20)	0.96 (0.80-1.14)	0.94 (0.75-1.17)	0.02
LDL-C (mmol/L)	2.13 (1.61-2.70)	2.26 (1.77-2.88)	2.24 (1.79-2.84)	2.27 (1.79-2.81)	<0.01
FPG (mmol/L)	5.20 (4.60-5.90)	5.30 (4.60-6.00)	5.40 (4.80-6.30)	5.60 (4.80-6.90)	<0.01
NT-proBNP (pmol/L)	3100.00 (1716.50-5139.00)	3678.00 (1846.50-6008.50)	3825.00 (1892.00-6473.00)	4241.00 (2052.07-7270.25)	<0.01
30-day mortality (n,%)	16 (2.32%)	23 (3.33%)	36 (5.21%)	128 (18.50%)	<0.01

ADHF, acute decompensated heart failure; AISI, Aggregate Index of Systemic Inflammation; CHD, coronary heart disease; NYHA, New York Heart Association; LVEF: left ventricular ejection fraction; TG, triglyceride; TC, total cholesterol; HDL-C, high-density lipoprotein cholesterol; LDL-C, low-density lipid cholesterol; Cr, creatinine; WBC, white blood cell count; RBC, red blood cell count; PLT, platelet count; ALT, alanine aminotransferase; AST, aspartate aminotransferase; NT-proBNP, N-Terminal Pro-Brain Natriuretic Peptide; UA, uric acid; FPG, fasting plasma glucose.

### Association between AISI quartiles, LnAISI, and 30-day mortality in ADHF patients

During a median 30-day follow-up, 203 ADHF patients (7.34%) died, with mortality rates increased across AISI quartiles (2.32%, 3.33%, 5.21%, and 18.50%, respectively). As shown in **Figure 2**, Kaplan-Meier survival analysis revealed significantly worse survival outcomes in the highest AISI quartile (Q4) compared to the lower quartiles (log-rank test *P* < 0.0001), with a substantially elevated 30-day mortality rate.

The associations between AISI quartiles, LnAISI, and 30-day mortality in ADHF patients are presented in **Table 2**. Notably, LnAISI consistently demonstrated a positive association with 30-day mortality in ADHF patients from crude model to adjusted model. In the fully adjusted model, each one-unit increase in LnAISI was associated with a 75% higher 30-day mortality risk in ADHF patients (HR: 1.75, 95% CI: 1.51-2.04). ADHF patients in the highest AISI quartile exhibited a 210% elevated risk of 30-day mortality compared to those in the lowest quartile (HR: 3.10, 95% CI: 1.62-5.94). Overall, the association between AISI and 30-day mortality in ADHF patients maintained a consistent positive trend (*P*-trend < 0.01).

**Table 2:** Multivariable Cox regression analysis of the association between AISI and 30-day mortality in patients with ADHF.

Independent variable	Hazard ratios (95% confidence interval)			
	Unadjusted Model	Model I	Model II	Model III
LnAISI	2.16 (1.97, 2.36)	2.16 (1.96, 2.39)	2.03 (1.81, 2.28)	1.75 (1.51, 2.04)
AISI quartiles				
Q1	1.0	1.0	1.0	1.0
Q2	1.44 (0.76, 2.73)	1.39 (0.74, 2.64)	1.10 (0.56, 2.16)	0.74 (0.33, 1.68)
Q3	2.28 (1.26, 4.10)	2.15 (1.19, 3.89)	1.99 (1.08, 3.65)	1.89 (0.95, 3.75)
Q4	8.67 (5.15, 14.58)	7.64 (4.53, 12.90)	5.30 (3.06, 9.17)	3.10 (1.62, 5.94)
*P*-trend	<0001	<0.01	<0.01	<0.01

AISI, Aggregate Index of Systemic Inflammation; ADHF, acute decompensated heart failure.

Model I adjusted for gender, age, hypertension, diabetes, stroke and CHD.

Model II adjusted for model I + NYHA classification, drinking status, smoking status, LVEF.

Model III adjust for, Model II + RBC, AST, Cr, UA, TC, TG, TC, HDL-C, LDL-C, FPG and NT-proBNP.

### Dose-response relationship between AISI, LnAISI, and 30-day mortality in ADHF patients

**Figure 3:**
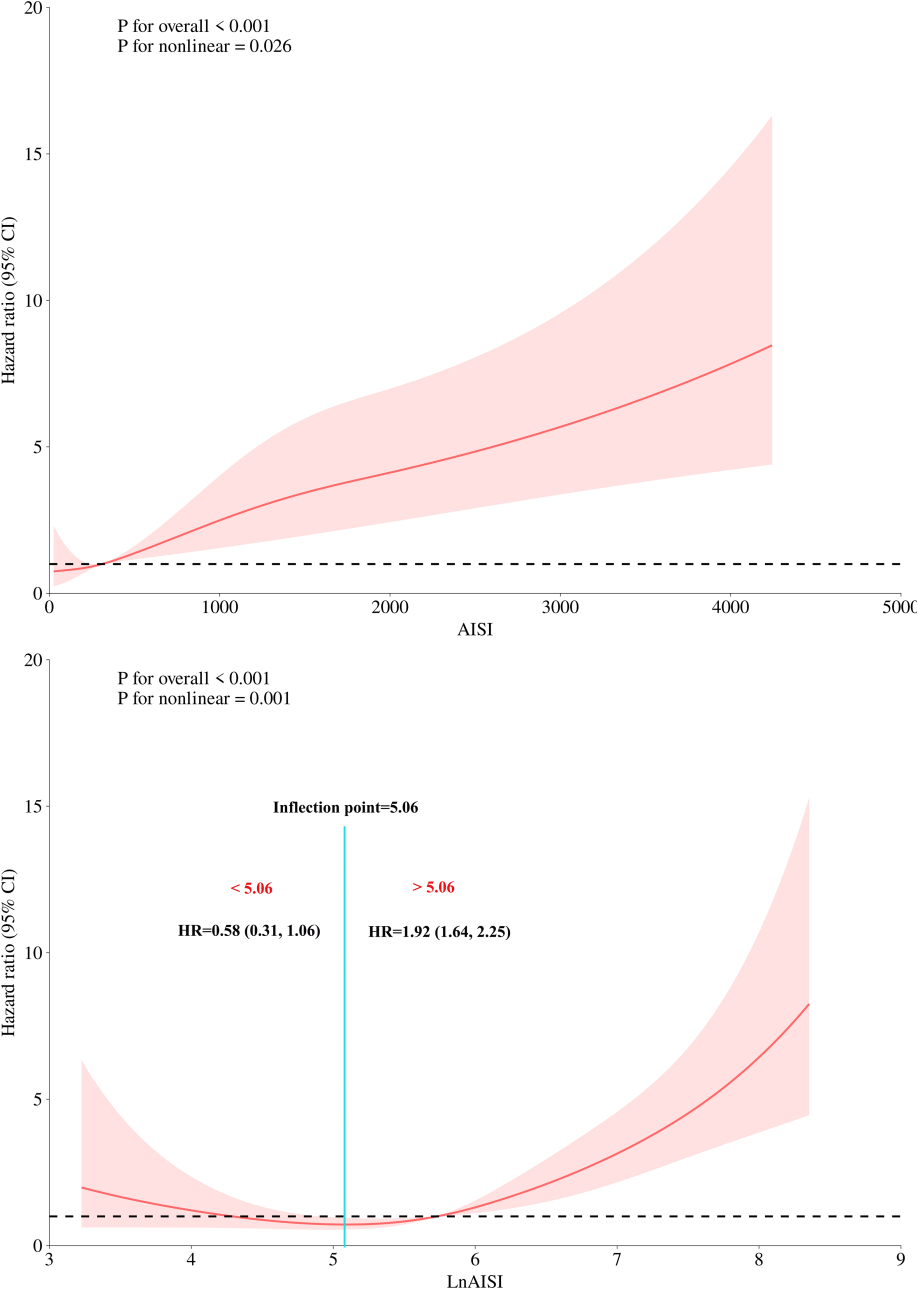
Dose-response relationship between AISI (and Ln AISI) and 30-day all-cause mortality analyzed by restricted cubic spline (4 knots). Adjusted for demographics, comorbidities, laboratory markers, and cardiac function indicators (see Methods for details). The association between the 30-day mortality rate and AISI (and LnAISI) followed a U-shaped curve, with the lowest 30-day mortality risk in ADHF patients observed at an AISI of approximately 5.06, LnAISI showed an inverse association with 30-day mortality in ADHF patients when values were below 5.06 (HR: 0.58, 95% CI: 0.31-1.06), while a positive association was observed above this threshold (HR: 1.92, 95% CI: 1.64-2.25). AISI, Aggregate Index of Systemic Inflammation; ADHF, acute decompensated heart failure; HR, Hzard ratio; CI, confidence interval.

Based on Model III, we further modeled the dose-response relationship between AISI, LnAISI, and 30-day mortality in ADHF patients using RCS. As shown in **Figure 3**, after full adjustment for potential confounders, the analysis revealed a U-shaped dose-response relationship between AISI, LnAISI, and 30-day mortality in ADHF patients (*P* for nonlinearity <0.05). Collectively, both extremely low and high levels of AISI or LnAISI were associated with an increased risk of 30-day mortality risk in ADHF patients. We estimated the inflection point at which the association between LnAISI and 30-day mortality changes as 5.06 using a recursive algorithm (**Table 3**). Piecewise Cox regression analysis further revealed that LnAISI demonstrated an inverse association with 30-day mortality in ADHF patients when below 5.06 (HR: 0.58, 95% CI: 0.31-1.06), whereas a positive association was observed beyond this threshold (HR: 1.92, 95% CI: 1.64-2.25).

**Table 3:** The result of the two-piecewise Cox regression model.

Independent variable	Hazard ratios (95% confidence interval)
Fitting model by two-piecewise cox regression	
The inflection point of AISI	5.06
< 5.06	0.58 (0.31, 1.06)
> 5.06	1.92 (1.64, 2.25)
Log likelihood ratio test	<0.01

AISI, Aggregate Index of Systemic Inflammation; ADHF, acute decompensated heart failure.

Adjusted for gender, age, hypertension, diabetes, stroke, CHD, NYHA classification, drinking status, smoking status, LVEF, RBC, AST, Cr, UA, TC, TG, TC, HDL-C, LDL-C, FPG, and NT-proBNP.

### Subgroup analysis

**Figure 4:**
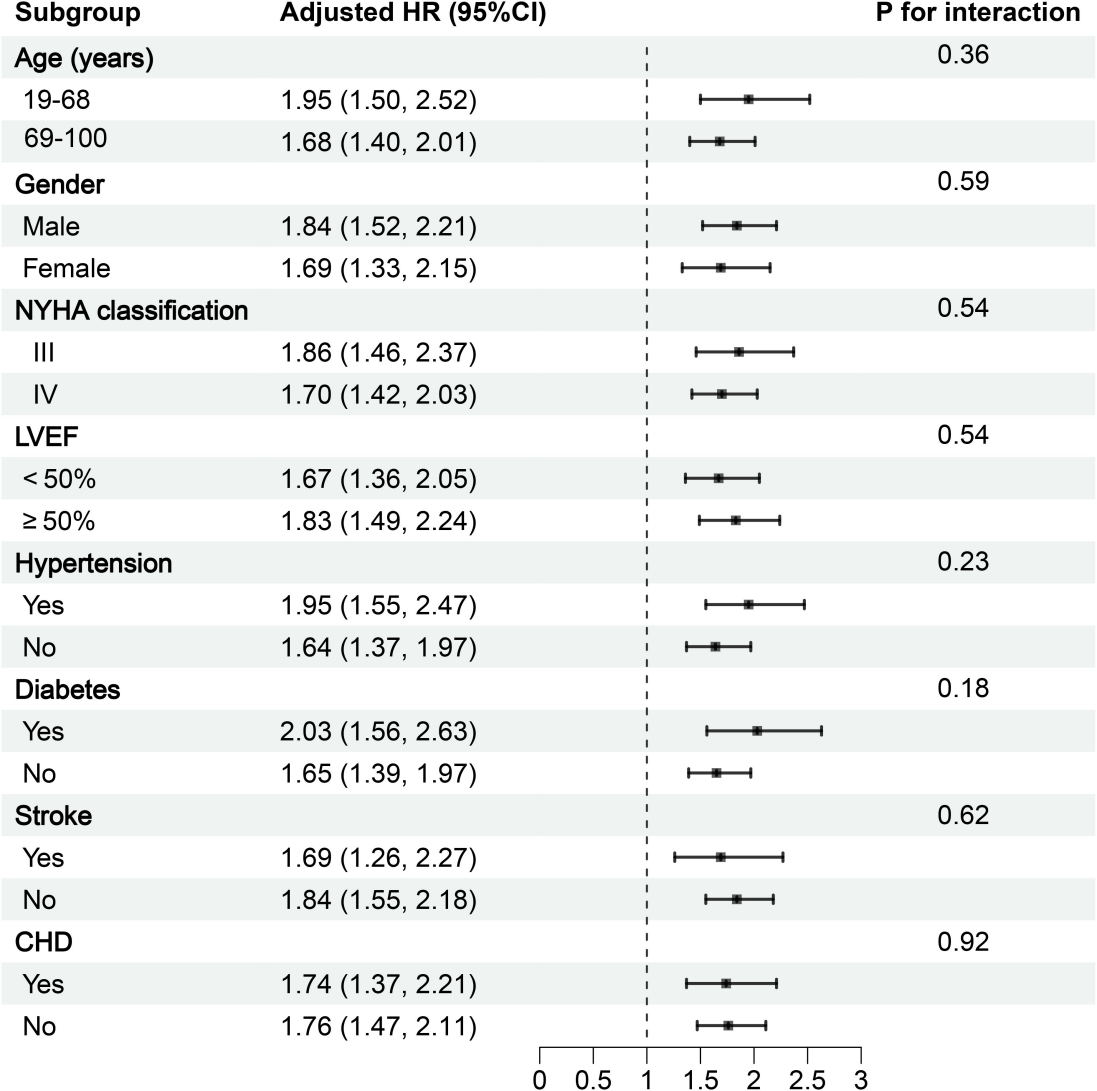
Forest plot of subgroup analysis on the association between AISI and 30-day mortality in ADHF patients. ADHF, acute decompensated heart failure; AISI, Aggregate Index of Systemic Inflammation; NYHA, New York Heart Association; LVEF, left ventricular ejection fraction; CHD, coronary heart disease; All models were adjusted for covariates in Model III (**Table 2**), excluding the stratified variable itself.

Based on the fully adjusted model, we conducted exploratory subgroup analyses to examine the association between LnAISI and 30-day mortality risk in ADHF patients across subgroups defined by gender, age, LVEF, NYHA classification, and comorbid hypertension, diabetes, stroke, and CHD. Detailed results are presented in **Figure 4**. In subgroup analyses, we observed relatively higher mortality risk associated with LnAISI in patients aged <69 years, males, those with NYHA Class III status, and individuals with comorbid hypertension/stroke/diabetes. However, interaction tests revealed no significant differences across all subgroups (all *P*-interaction > 0.05). These findings suggested that the association between LnAISI and 30-day mortality was relatively robust across clinical subgroups.

### Receiver operating characteristic analysis

**Figure 5:**
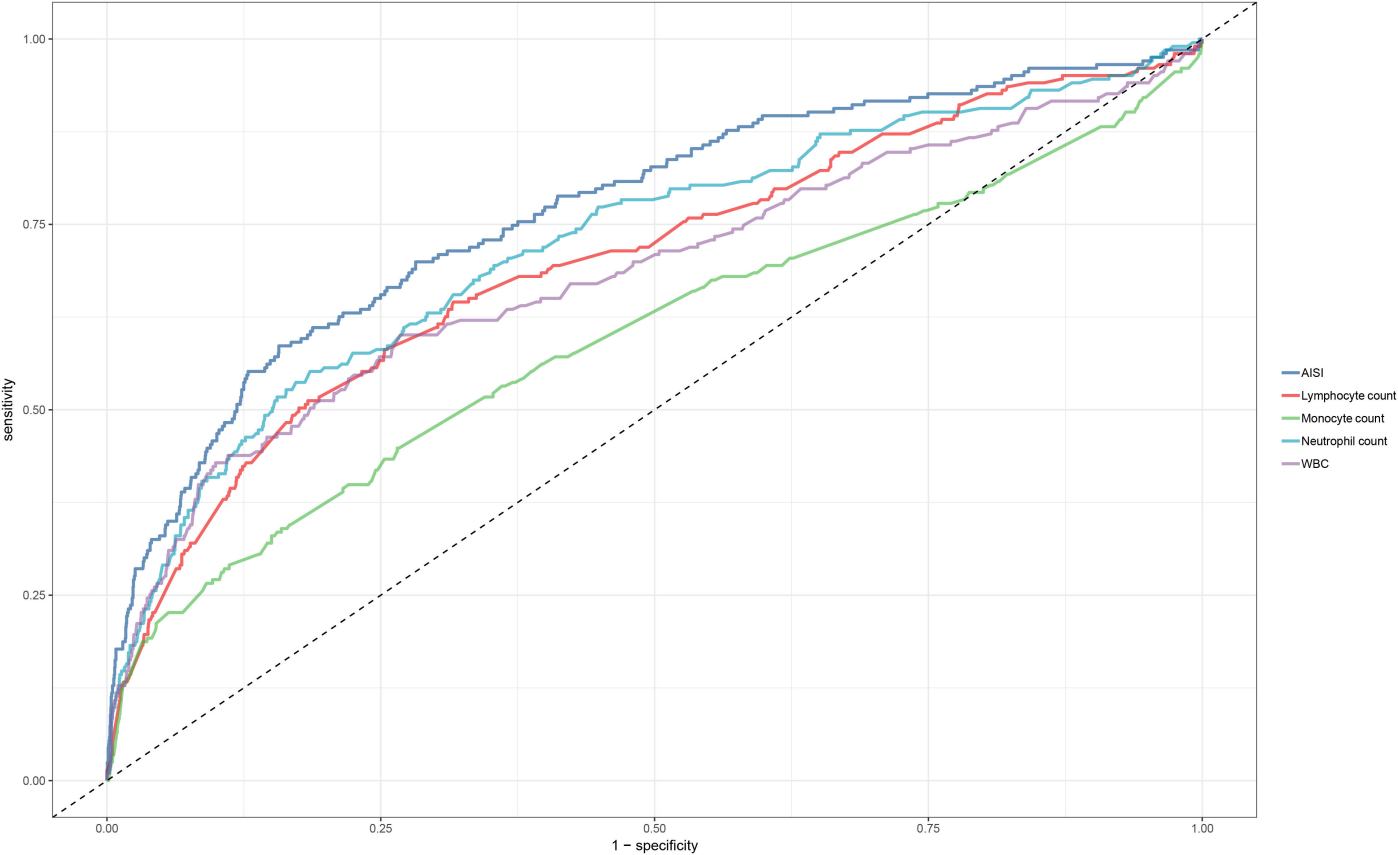
ROC curve analysis of AISI and conventional inflammatory markers (neutrophils, lymphocytes, monocytes, WBC) in predicting 30-day mortality in ADHF patients. ADHF, acute decompensated heart failure; AISI, Aggregate Index of Systemic Inflammation; ROC, Receiver operating characteristic; WBC, white blood cell count.

Regarding the predictive value of inflammatory markers for short-term prognosis in ADHF patients, **Figure 5** provides a systematic comparison of AISI and several commonly used inflammatory indicators (including neutrophil count, lymphocyte count, monocyte count, and WBC count) for predicting 30-day mortality. Among these markers, AISI demonstrated the highest discriminative ability (AUC = 0.77), significantly outperforming neutrophil count (AUC = 0.72), lymphocyte count (AUC = 0.70), monocyte count (AUC = 0.60), and WBC (AUC = 0.68) (**Table 4**, all DeLong’s test *P* < 0.05). Further analysis identified optimal thresholds of 925.44 for AISI and 6.83 for LnAISI in predicting 30-day mortality risk in ADHF patients.

**Table 4:** ROC analysis of AISI and various commonly used inflammatory indicators on the predictive value of 30-day mortality in ADHF patients.

Variable	AUC	95%CI low	95%CI upp	Best threshold	Specificity	Sensitivity
AISI	0.77	0.73	0.81	925.44	0.84	0.59
LnAISI	0.77	0.73	0.81	6.83	0.84	0.59
WBC*	0.68	0.64	0.73	7.70	0.73	0.60
Neutrophil count*	0.72	0.68	0.76	6.60	0.81	0.55
Lymphocyte count*	0.70	0.66	0.74	0.84	0.68	0.65
Monocyte count*	0.60	0.55	0.64	0.61	0.74	0.45

AISI, Aggregate Index of Systemic Inflammation; ADHF, acute decompensated heart failure; WBC, white blood cell count; ROC, Receiver operating characteristic; AUC, area under the curve. *P<0.05, compare with AISI.

### Incremental predictive performance of AISI added to NT-proBNP or LVEF models for mortality assessment

The integration of AISI into the NT-proBNP or LVEF models significantly enhanced the predictive performance for 30-day mortality (**Supplementary Table 4**). Specifically, the AUC of the NT-proBNP model increased from 0.66 to 0.81 (DeLong test *P* < 0.01), while the LVEF model’s AUC rose from 0.50 to 0.76 (DeLong test *P* < 0.01), demonstrating that AISI provided substantial incremental predictive value for both models.

### Sensitivity analysis

As shown in **Table 5**, sensitivity analyses excluding potentially frail subgroups yielded results consistent with the primary findings. After adjusting for potential reverse causality, the association pattern between AISI and outcomes in ADHF patients remained unchanged. Exclusion of patients with baseline pulmonary infections demonstrated no substantial change in the association strength between AISI and ADHF prognosis, further supporting the robustness of primary conclusions. Finally, to assess potential biases from missing data, we conducted multiple imputation, and key findings from the complete dataset analysis remained highly consistent with initial analyses, validating the reliability of our conclusions. Collectively, the robustness of our findings was systematically confirmed through a series of complementary analyses, including subgroup assessments, temporal sensitivity analyses, and data integrity verification, across diverse clinical scenarios.

**Table 5:** Sensitivity analysis.

Independent variable	Hazard ratios (95% confidence interval)			
	Sensitivity-1	Sensitivity-2	Sensitivity-3	Sensitivity-4
LnAISI	1.70 (1.44, 2.00)	2.06 (1.72, 2.46)	2.50 (1.92, 3.24)	1.73 (1.54, 1.95)
AISI quartiles				
Q1	Ref	Ref	Ref	Ref
Q2	0.69 (0.30, 1.62)	0.77 (0.27, 2.22)	0.56 (0.13, 2.40)	1.18 (0.62, 2.24)
Q3	1.52 (0.75, 3.09)	2.16 (0.90, 5.17)	1.90 (0.62, 5.82)	1.68 (0.93, 3.06)
Q4	2.67 (1.38, 5.17)	4.47 (1.97, 10.15)	6.47 (2.30, 18.20)	3.76 (2.18, 6.49)
*P*-trend	<0.01	<0.01	<0.01	<0.01

AISI, Aggregate Index of Systemic Inflammation.

Note 1, Adjusted for sex, age, hypertension, diabetes, stroke, CHD, NYHA classification, drinking status, smoking status, LVEF, monocyte count, RBC, PLT, AST, Cr, UA, TC, TG, HDL-C, LDL-C, FPG and NT-proBNP.

Note 2, Hypertension, diabetes, Cerebral stroke and CHD were not adjusted in Sensitivity-1.

## Discussion

The evaluation of novel inflammatory indices is essential for early risk stratification in ADHF. To the best of our knowledge, this study is the first to investigate the association between AISI and prognosis in ADHF among Chinese patients. Our findings demonstrate that both extremely low and elevated level of AISI are significantly associated with adverse outcomes in ADHF, exhibiting a U-shaped, curvilinear relationship. Furthermore, compared with traditional inflammatory markers, AISI showed superior predictive performance for short-term prognosis in ADHF patients and offered additional incremental predictive value when integrated with NT-proBNP or LVEF models for mortality assessment. These findings suggest that AISI may serve as a useful prognostic marker for early risk stratification in the management of ADHF.

ADHF refers to a clinical syndrome characterized by either rapid deterioration of cardiac function in patients with pre-existing chronic HF or *de novo* onset of cardiac dysfunction, typically precipitated by identifiable triggers. It manifests with severe symptoms and signs that require urgent medical intervention. The pathophysiological core of ADHF involves an abrupt decline in cardiac pumping capacity over a short period, during which compensatory mechanisms fail to sustain adequate circulatory function, leading to fluid retention and impaired tissue perfusion (1–3). Although the exact pathogenesis of ADHF has not been fully elucidated (10), current evidence indicates a strong association between ADHF onset and significant upregulation of inflammatory pathways (11–13). Notably, these inflammatory processes have been demonstrated to exacerbate disease progression in ADHF (10, 40, 41). During ADHF exacerbation, circulating levels of various pro-inflammatory cytokines and mediators are markedly elevated, contributing to further disease progression. This notion is supported by accumulating clinical evidence, which underscores the significant prognostic value of early inflammatory biomarker detection in predicting adverse outcomes associated with ADHF (42–45). Notably, dynamic changes in inflammatory markers often precede alterations in conventional biomarkers such as NT-proBNP (14), suggesting that early inflammatory monitoring may not only enhance prognostic stratification but also provide a critical window for timely clinical intervention.

As an emerging tool for systemic inflammation assessment, the AISI has gained significant research attention in recent years by integrating multi-dimensional inflammatory parameters. Furthermore, growing evidence-based medical research highlights its unique advantages in risk stratification and prognostic evaluation across diverse disease contexts spanning multiple disciplines, offering a novel biomarker option for clinical decision-making (19–29). Given the involvement of inflammatory mechanisms in the pathogenesis of multiple CVDs, the potential applications of AISI in this field have generated considerable research enthusiasm (30–36). Emerging evidence indicates a strong association between this biomarker and both CVD risk and prognostic outcomes. To date, multiple international studies have reported associations between AISI and HF (30, 46). Furthermore, growing evidence supports the critical prognostic value of AISI in the management of CVDs (35, 36). In the current study, we evaluated the association between AISI and 30-day mortality outcomes among ADHF patients in Jiangxi, China. Our analysis revealed that ADHF patients in the highest AISI quartile (Q4) had a 3.1-fold increased risk of all-cause death within 30 days compared to those in the lowest quartile (Q1). Building upon previous research on HF risk assessment (30, 46), the present study provides further evidence supporting the prognostic utility of AISI in HF patients. Compared to its application in assessing HF incidence risk, AISI appears to offer more substantial predictive value for HF prognosis. Moreover, these results expand the clinical applications of AISI in CVD prognosis assessment. In light of existing evidence demonstrating the prognostic value of AISI in patients with hypertension and acute myocardial infarction (35, 36), our findings suggest that AISI may be particularly well-suited for short-term risk assessment in CVD management.

Our findings highlighted that the association between AISI and 30-day mortality in ADHF patients may be nonlinear. Notably, we observed a U-shaped relationship between AISI and 30-day mortality in this population: both extremely low and excessively high AISI levels act as risk factors for elevated 30-day mortality. Similar nonlinear associations have been reported in several recent AISI-related studies (19, 24, 29, 31), several of which specifically evaluated the inflection point for LnAISI-clinical outcome associations: Yin et al. identified a special “U-shaped” association between LnAISI and RA risk, calculating a curve inflection point at 5.70, while Huang et al. calculated an inflection point of 5.20 for the LnAISI-CKD association (19, 24). In the current study, a recursive algorithm was applied to identify the inflection points in the association between the LnAISI and 30-day mortality among ADHF patients, revealing a threshold at 5.06. Below this value, LnAISI was inversely associated with mortality, whereas a positive association was observed above 5.06. This threshold demonstrates remarkable consistency with previous reports by Yin et al. and Huang et al. (19, 24), collectively indicating that maintaining LnAISI values within the 5.0–6.0 range may facilitate risk control for both disease severity and adverse prognosis. The association between elevated AISI levels and increased mortality risk is readily understandable, primarily attributable to direct inflammatory storm-mediated damage. Regarding the increased mortality observed at low AISI levels, we postulate that it may be attributed to mechanisms including lymphocyte depletion, immunosuppression, advanced cachexia, and iatrogenic factors: (i) While low AISI may indicate a relative predominance of lymphocytes, however, in advanced HF this could signify an “immune exhaustion” state, where persistent inflammatory stimulation leads to T-cell exhaustion and impaired capacity to regulate inflammatory responses (47, 48). (ii) In end-stage HF patients, intestinal congestion-induced malabsorption and reduced hepatic synthetic function contribute to muscle wasting and lipolysis (49–52); meanwhile, the body may catabolize immunoglobulins and lymphocyte proteins for energy, further exacerbating immune exhaustion (53, 54). (iii) Intracellular ion homeostasis is crucial for maintaining normal cellular function. In end-stage HF patients, prolonged use of diuretics and digoxin may lead to chronic hypokalemia, which causes suppression of T-cell function (55, 56). In addition, prolonged use of β-blockers may lead to partial suppression of immune function: Theoretically, β-adrenergic receptors are expressed on both innate immune cells (e.g., neutrophils, macrophages) and adaptive immune cells (e.g., T lymphocytes, B lymphocytes, natural killer cells); these receptors may exert inhibitory effects on immune cells, and chronic stimulation of these receptors could result in immune dysfunction (57). Overall, the RCS-based findings in this study carry dual clinical significance: First, the dose-response relationship curves visually demonstrate the nonlinear association patterns between AISI/LnAISI and 30-day mortality risk in ADHF patients, revealing both overall associations and stage-specific dynamic changes. Second, the nadir of this U-shaped curve (LnAISI = 5.06) has significant clinical value: At the risk prognostic level, this threshold may serve as the optimal cutoff for minimal short-term mortality risk in ADHF risk stratification.

The underlying mechanisms linking AISI to short-term adverse outcomes in ADHF remain unclear, though preliminary insights may be gleaned through analysis of the immune cell components that comprise AISI: (1) Activated neutrophils mediate the degranulation-dependent release of large quantities of proteolytic enzymes, which directly degrade the cardiac extracellular matrix and induce cardiomyocyte apoptosis/necrosis. This pathological process not only exacerbates cardiac systolic dysfunction but also amplifies inflammatory cascades through the release of damage-associated molecular patterns, ultimately contributing to poor prognosis in ADHF (58, 59). Notably, neutrophil-derived inflammatory factors can also induce lymphocyte apoptosis (60). Furthermore, the concurrent presence of persistent neutrophilia and lymphopenia often serves as a clinical indicator of extensive myocardial injury and elevated short-term mortality risk in HF patients (17, 61). (2) Monocytes play a significant role in myocardial injury, through the following mechanisms (62):: (i) Direct participation in inflammatory infiltration and immune activation; (ii) Promotion of myocardial remodeling, including hypertrophy and fibrosis; (iii)Induction of cell death programs. Additionally, the inflammatory mediators they secrete may further accelerate lymphocyte apoptosis, and this “cross-talk” between immune cells significantly exacerbates the progression of HF (60, 63, 64). (3) In addition to being influenced by inflammatory mediators mediated by other immune cells (60, 63, 64), lymphocytes are also affected by the clinical symptoms of HF. Studies have shown that visceral congestion in HF can trigger a bidirectional vicious cycle of “lymphocyte loss-myocardial injury” (65, 66): On one hand, intestinal congestion leads to abnormal lymphocyte loss via the mesenteric lymphatic system; on the other hand, this immunocyte depletion accelerates myocardial remodeling by weakening anti-inflammatory defenses, forming a self-amplifying pathophysiological loop. (4) Although PLTs are not typical immune cells, their activation is closely associated with poor prognosis in HF (67). They primarily exert their effects through the following pathways (68, 69): (i) hemodynamic disturbances; (ii) vascular dysfunction; and (iii) regulation of chemokine networks, collectively serving as key drivers of HF progression. (4) Another critical consideration is the crosstalk between neurohormonal pathways and the immune system during acute HF exacerbation. Studies indicate that activated neurohumoral mechanisms modulate immune cells in HF (70, 71): From an AISI perspective, evidence shows that Renin-Angiotensin-Aldosterone System activation in HF patients leads to angiotensin II-mediated regulation of macrophage phenotypes by promoting M2 macrophage polarization; this stimulation may influence Th1/Th2 lymphocyte balance, induce lymphocyte apoptosis, and ultimately reduce lymphocyte counts (70, 72). Moreover, neurohormonal activation can trigger inflammatory responses by activating the nuclear factor-κB inflammatory pathway and upregulating pro-inflammatory mediators, including tumor necrosis factor-α and monocyte chemoattractant protein-1 (73, 74). On the other hand, neurohormonal activation increases plasma cortisol levels and catecholamine release, leading to downregulated lymphocyte differentiation and proliferation, followed by enhanced lymphocyte apoptosis (75, 76). Based on these findings, we recommend that early risk stratification of ADHF patients using AISI should also account for the promoting effects of neurohormonal pathways.

The strengths of this study include the novelty of the research topic, the ease of obtaining study variables, and the support from a large-scale cohort study, which collectively confer innovative clinical significance and generalizability to the AISI in short-term prognostic assessment for ADHF patients. Furthermore, through multi-faceted validation analyses, including subgroup analysis, temporal sensitivity analysis, and data integrity tests, we systematically validated the robustness of our findings across diverse clinical scenarios.

This study also has several limitations that warrant consideration: (1) As a non-interventional observational study, its design inherently precludes evaluation of post-admission treatment benefits for ADHF patients (77) and causal inferences about therapeutic efficacy. Consequently, conclusions are limited to disease natural history and biomarker associations. (2) This study focuses on the predictive value of AISI levels at admission for short-term mortality prognosis in ADHF patients, without exploring the association between in-hospital AISI changes and clinical outcomes. Future research needs to employ continuous biomarker monitoring to further clarify the incremental prognostic value of temporal AISI evolution patterns. (3) Although this study employed multivariable adjustment and sensitivity analyses to rigorously control for known confounders, the inherent limitations of observational research preclude the complete exclusion of residual confounding effects. (4) Given that participants were primarily recruited from Jiangxi Province in southern China, the generalizability of our findings to northern China and different ethnic populations requires further validation.

## Conclusion

This population-based retrospective cohort study is the first to evaluate the predictive value of AISI for short-term prognosis in ADHF patients within the Chinese population. Compared with conventional inflammatory markers, AISI demonstrates superior predictive performance for 30-day mortality. These findings have important clinical implications, as they may facilitate optimized prevention strategies for adverse outcomes in ADHF and enable early risk stratification through personalized assessment of individual ADHF patients.

## Data Availability

The raw data supporting the conclusions of this article will be made available by the authors, without undue reservation.
