# Supplementary material for: Aggregate index of systemic inflammation as a novel prognostic biomarker in Chinese patients with acute decompensated heart failure: a population-based real-world study
Source: Front Endocrinol (Lausanne). 2025 Aug 13;16:1627821. doi: 10.3389/fendo.2025.1627821 (PMC12380547; doi:10.3389/fendo.2025.1627821)
Supplement: Supplementary file 1 [file DataSheet1.docx]

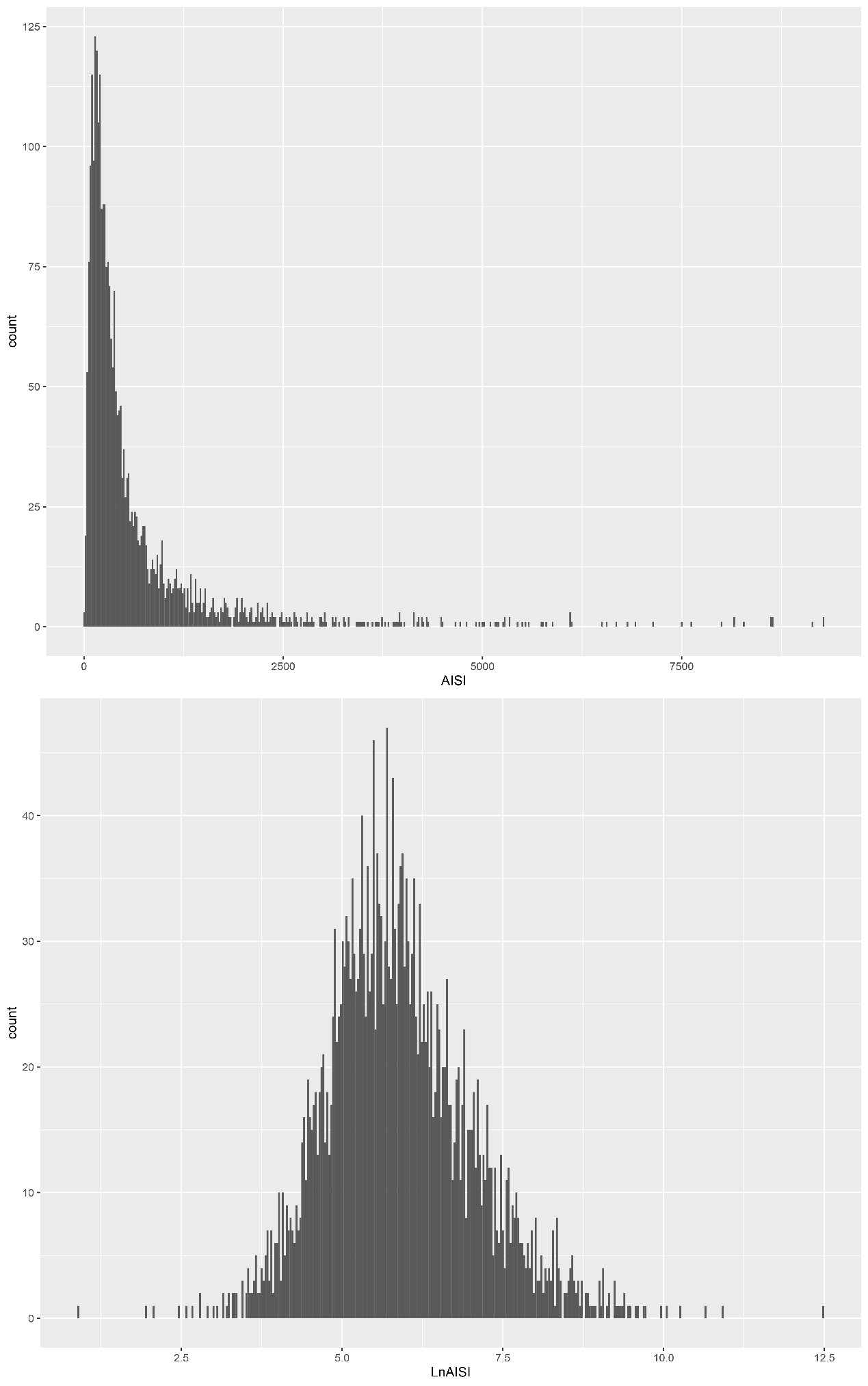


Supplementary Figure 1: The distribution of AISI and LnAISI. AISI: Aggregate Index of Systemic Inflammation;


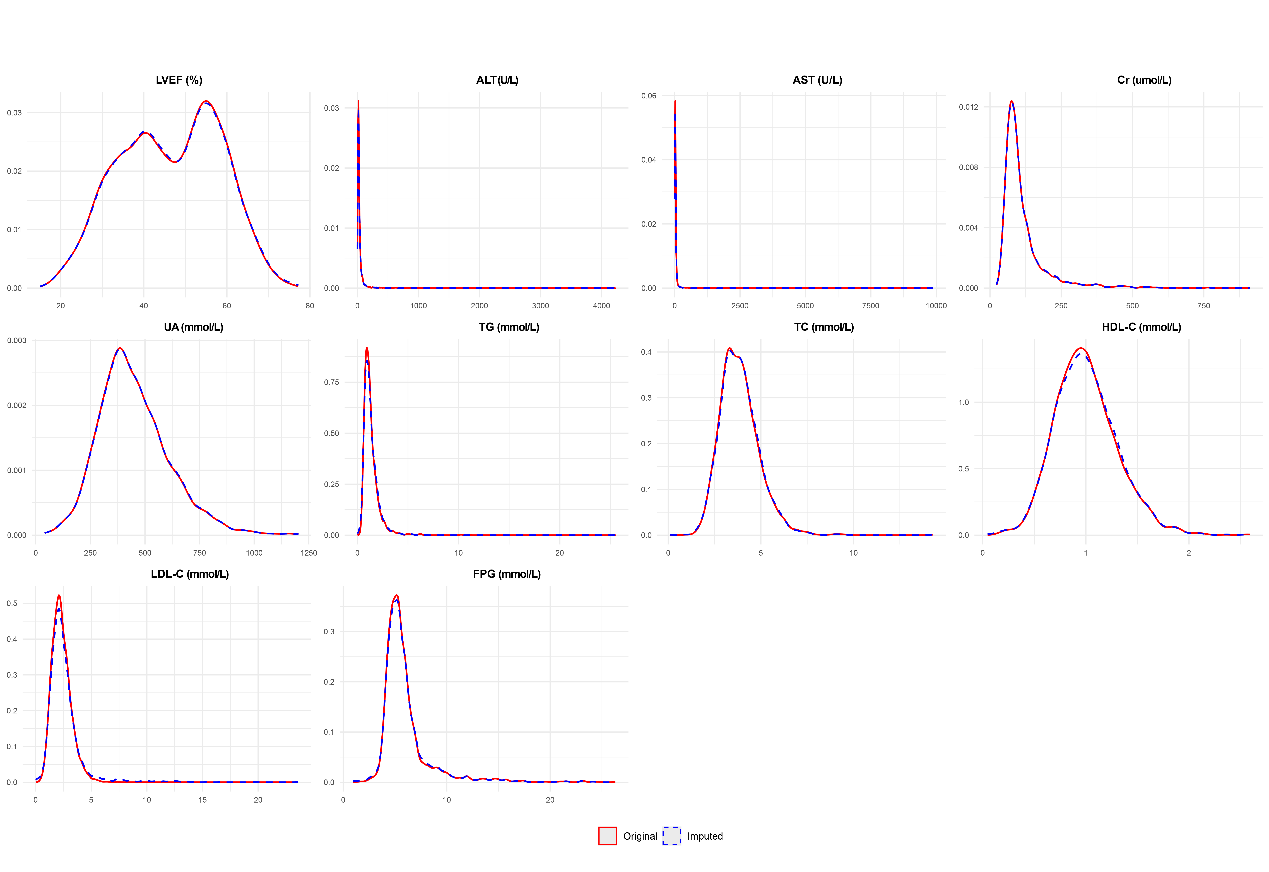


**Supplementary Figure 2:** Density plots of missing data distribution before and after imputation. LVEF: left ventricular ejection fraction; TG: triglyceride; TC: total cholesterol; HDL-C: high-density lipoprotein cholesterol; LDL-C: low-density lipid cholesterol; Cr: creatinine; ALT: alanine aminotransferase; AST: aspartate aminotransferase; UA: uric acid; FPG: fasting plasma glucose.
