# Supplementary material for: Aggregate index of systemic inflammation as a novel prognostic biomarker in Chinese patients with acute decompensated heart failure: a population-based real-world study
Source: Front Endocrinol (Lausanne). 2025 Aug 13;16:1627821. doi: 10.3389/fendo.2025.1627821 (PMC12380547; doi:10.3389/fendo.2025.1627821)
Supplement: Supplementary file 2 [file DataSheet2.docx]

Supplementary Table 1: The missing number and rate of covariates.

|  | Non- Missing | Missing |
| --- | --- | --- |
| Age | 2765 | 0 |
| Gender | 2765 | 0 |
| Hypertension | 2765 | 0 |
| Diabetes | 2765 | 0 |
| Stroke | 2765 | 0 |
| CHD | 2765 | 0 |
| NYHA classification | 2765 | 0 |
| Drinking status | 2765 | 0 |
| Smoking status | 2765 | 0 |
| LVEF | 2632 | 133 |
| WBC | 2765 | 0 |
| Neutrophil count | 2765 | 0 |
| Lymphocyte count | 2765 | 0 |
| Monocyte count | 2765 | 0 |
| RBC | 2765 | 0 |
| PLT | 2765 | 0 |
| ALT | 2705 | 60 |
| AST | 2714 | 51 |
| Cr | 2727 | 38 |
| UA | 2726 | 39 |
| TG | 2410 | 355 |
| TC | 2410 | 355 |
| HDL-C | 2410 | 355 |
| LDL-C | 2410 | 355 |
| FPG | 2630 | 135 |
| NT-proBNP | 2765 | 0 |

Abbreviations as in Table 1.

Supplementary Table 2: Comparison of baseline data between people with missing data and people without missing data

|  | Missing data | Non-missing data | *P*-value |
| --- | --- | --- | --- |
| LVEF | 47.91 (11.73) | 46.05 (12.09) | 0.003 |
| ALT | 20.00 (14.00-35.00) | 22.00 (14.00-39.00) | 0.139 |
| AST | 26.50 (20.00-41.00) | 26.00 (20.00-39.00) | 0.453 |
| Cr | 89.00 (67.00-129.00) | 90.00 (71.00-125.25) | 0.256 |
| UA | 420.00 (332.25-529.75) | 429.00 (342.00-544.00) | 0.237 |
| TG | 1.14 (0.93-1.53) | 1.14 (0.86-1.54) | 0.626 |
| TC | 3.69 (3.21-4.37) | 3.76 (3.11-4.44) | 0.951 |
| HDL-C | 1.00 (0.82-1.20) | 0.97 (0.80-1.17) | 0.469 |
| LDL-C | 2.24 (1.74-2.80) | 2.22 (1.75-2.81) | 0.939 |
| FPG | 6.25 (2.72) | 5.92 (2.41) | 0.010 |

Abbreviations as in Table 1.

Supplementary Table 3: Collinearity diagnostics steps.

|  | VIF | | |
| --- | --- | --- | --- |
|  | Step 1 | Step 2 | Step 3 |
| AISI | 1.1 | 1.1 | 1.1 |
| Age | 1.4 | 1.4 | 1.4 |
| Gender | 1.3 | 1.3 | 1.3 |
| Hypertension | 1.2 | 1.2 | 1.1 |
| Diabetes | 1.4 | 1.4 | 1.4 |
| Stroke | 1.1 | 1.1 | 1.1 |
| CHD | 1.2 | 1.2 | 1.2 |
| NYHA classification | 1.1 | 1.1 | 1.1 |
| Drinking status | 1.5 | 1.5 | 1.5 |
| Smoking status | 1.6 | 1.6 | 1.6 |
| LVEF | 1.2 | 1.2 | 1.2 |
| WBC | 337.9 | NA | NA |
| Neutrophil count | 308.2 | 1.7 | 1.7 |
| Lymphocyte count | 15.5 | 1.5 | 1.5 |
| Monocyte count | 3.7 | 1.5 | 1.5 |
| RBC | 1.4 | 1.4 | 1.4 |
| PLT | 1.3 | 1.3 | 1.3 |
| ALT | 7.4 | 7.4 | NA |
| AST | 7.2 | 7.1 | 1.1 |
| Cr | 1.5 | 1.5 | 1.5 |
| UA | 1.4 | 1.4 | 1.4 |
| TG | 1.5 | 1.5 | 1.5 |
| TC | 1.8 | 1.8 | 1.8 |
| HDL-C | 1.6 | 1.6 | 1.6 |
| LDL-C | 1 | 1 | 1 |
| FPG | 1.4 | 1.4 | 1.4 |
| NT-proBNP | 1.3 | 1.3 | 1.3 |

Abbreviations as in Table 1.

Supplementary Table 4: Evaluating the incremental prognostic value of adding the AISI to the NT-proBNP model or LVEF model for predicting 30-day mortality.

| Model | AUC (95 CI) | *Delong P* value |
| --- | --- | --- |
| NT-proBNP Model | 0.66 (0.62, 0.70) |  |
| NT-proBNP + AISI | 0.81 (0.78, 0.84) | <0.01 |
| LVEF Model | 0.50 (0.45, 0.54) |  |
| LVEF + AISI | 0.76 (0.71, 0.80) | <0.01 |

Abbreviations: CI confidence interval; AISI: Aggregate Index of Systemic Inflammation; AUC: area under the curve; NT-proBNP: N-Terminal Pro-Brain Natriuretic Peptide; LVEF: left ventricular ejection fraction.
